# Research on the Process and Quality of Prepacked Braised Meat Products

**DOI:** 10.3390/foods14223937

**Published:** 2025-11-17

**Authors:** Mingxia Zhao, Lili Zhang, Li Liang, Shihao Sun, Shuxing Chen, Lishui Chen, Yuyu Zhang

**Affiliations:** 1 Food Laboratory of Zhongyuan, Beijing Technology and Business University, Beijing 100048, China; zhaozhang0823@163.com (M.Z.); zhanglili921116@163.com (L.Z.); 2 Food Laboratory of Zhongyuan, Luohe 462300, China; chengshuxing1@163.com (S.C.); chenlishui@zyfoodlab.cn (L.C.); 3 Beijing Life Science Academy, Beijing 102209, China; sunsh@ztri.com.cn; 4 Key Laboratory of Flavor Science of China General Chamber of Commerce, Beijing Technology and Business University, Beijing 100048, China

**Keywords:** processing method, physical and chemical analysis, shelf-life, analysis of volatile flavor substances

## Abstract

The processing technique critically determines the quality of prepackaged braised meat products. This study aimed to evaluate the effects of an innovative processing method against traditional methods on the product’s shelf-life and quality attributes. Results: no significant difference in shelf-life was observed between the experimental and control groups. However, the innovative method significantly improved product quality. The experimental group exhibited a redder and bluer color, significantly higher hardness (2–4 times, *p* < 0.01) and chewiness, alongside better moisture retention and meat yield. Sensory evaluation confirmed an overall preference for the experimental group (*p* < 0.05). Flavor profile analysis revealed a greater number and more stable retention of key flavor compounds (alcohols, ketones, and ethers) in the experimental group. The innovative processing method optimizes traditional techniques by significantly enhancing the physicochemical, textural, sensory, and flavor properties of prepackaged braised meat, without compromising shelf-life, providing a novel strategy for producing high-quality products.

## 1. Introduction

In the context of rapidly evolving consumer lifestyles, convenient and time-efficient food options have gained increasing popularity. Traditional Chinese prepackaged braised meat products are typically prepared from raw meat through blanching and subsequent cooking with diverse spices (e.g., star anise, cassia bark) and seasonings (e.g., salt, monosodium glutamate), resulting in a product known for its appealing color, rich flavor, fragrant aroma, and characteristic chewiness [1]. However, most such products are currently sold in bulk without effective sterilization. Due to their high protein content and water activity, they are susceptible to microbial contamination, which compromises both shelf stability and food safety [2]. While high-temperature and high-pressure sterilization can extend shelf life, it often leads to excessive heat exposure, causing deterioration of heat-sensitive nutrients, accelerated browning, and loss of the distinctive texture [3], thereby reducing consumer acceptance. Furthermore, traditional braising processes entail significant time and labor inputs, which constrains production efficiency. Thus, developing innovative processing techniques that balance shelf-life extension with the preservation of sensory, nutritional, and textural qualities while also reducing costs represents a critical challenge for the industry.

Current research remains limited in several key aspects: studies often focus on total flavor compounds or overall sensory evaluation, lacking a detailed analysis of the dynamic changes in critical flavor components or the interactions between spices and meat during sterilization. Moreover, claims regarding nutrient retention particularly protein content are frequently inadequately supported by statistical significance. Existing shelf-life assessments predominantly rely on fixed-temperature storage tests (e.g., 25 °C or 30 °C), failing to incorporate predictive models such as the Q10 coefficient method with multi-temperature gradients (e.g., 35 °C and 45 °C). This omission hinders the ability to scientifically project shelf life under diverse real-world storage conditions, such as refrigerated (10 °C) or ambient-temperature (30 °C) environments [4,5,6]. In response, this study introduces an innovative processing method that eliminates traditional prolonged braising. Instead, blanched raw meat is vacuum-sealed with spice seasoning packets and subjected to precisely controlled high-temperature sterilization, wherein the heating process simultaneously achieves cooking and preservation. This approach significantly shortens production cycles, enhances efficiency, and mitigates flavor and texture degradation. By integrating multi-temperature accelerated shelf-life testing and advanced flavor profiling, this research not only establishes a scientific basis for predicting shelf life under varying conditions but also provides new insights into dynamic flavor changes and nutrient retention, thereby offering a comprehensive solution for the production of high-quality, safe, and stable prepackaged braised meat products.

## 2. Materials and Methods

### 2.1. Materials

The principal ingredients employed in this study, beef shank meat (specifically from the flexor digitorum profundus and associated muscles, characterized by its high collagen and connective tissue content), boneless pork tenderloin (psoas major muscle), skinless, boneless chicken breast (Pectoralis major muscle), as well as spices and seasonings, were procured from the Dennis Fresh Supermarket in Luohe City, Henan Province, China. The vacuum bags (dimensions: 30 cm × 40 cm × 0.2 mm; material: PA + RCPP) utilized in the experiment were acquired from the official flagship store on Taobao.com. The experimental apparatus encompasses a vacuum packaging machine (model: DZ-400/2SB, manufacturer: Dongfeng Packaging, Wenzhou, China), an automatic autoclave (model: PHM-100, manufacturer: SunyOungster, Shanghai, China), a constant-temperature drying oven (model: DGH-9070B, manufacturer: OLABO, Jinan, China), an electronic balance (model: JA3203X, manufacturer: D&T, Tianjin, China), a texture analyzer (model: CTX, manufacturer: Brookfield, MA, USA), a colorimeter (model: RM200QC, manufacturer: x-rite, Grand Rapids, MI, USA), an automatic Kjeldahl nitrogen analyzer (model: K1160, manufacturer: Hanon, Jinan, China), and Top air solid phase microextraction and gas chromatography-mass spectrometry (HS-SPME-GC-MS) system (model: 8890 + 5977B, manufacturer: Agilent, Shanghai, China).

### 2.2. Preparation of the Experimental Group

**Figure 1:**
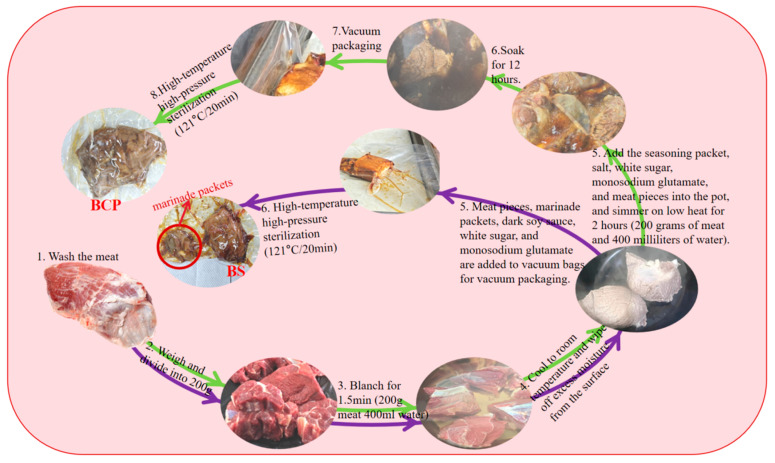
Processing flow diagram of the experimental group (green arrows) and the control group (purple arrows).

The beef shank meat (BS), boneless pork tenderloin (PS), and boneless chicken breast (CS)were cut into pieces weighing 200 g each and placed in cold water. Once the water reached a boiling state, the meat pieces were blanched for 1.5 min. After blanching, the meat was allowed to cool to room temperature. Subsequently, the cooled meat pieces, a spice mixture, and seasonings were placed into a vacuum bag and sealed using a vacuum sealer. Then, the sealed bag was placed in a pressure steam sterilizer and sterilized at 121 °C for 20 min. The specific procedure is depicted in Figure 1. Following the path indicated by the purple arrow, BS was used as the reference point.

### 2.3. Preparation of the Control Group

Beef shank meat (BCP), boneless pork tenderloin (PCP), and boneless chicken breast (CCP) were selected, cut into pieces weighing 200 g each, and placed in cold water. Once the water reached the boiling point, the meat was blanched for 1.5 min; the meat pieces were then transferred to cold water for a 30 min immersion. The meat pieces, spice packets, and seasonings were added to a 400-mL container. The contents were brought to a boil over high heat, then the heat was reduced, the contents were brought to a simmer and cooked for 2 h [7]. After cooking, the meat pieces were allowed to soak in the broth for an additional 12 h. Subsequently, the meat pieces were placed into vacuum bags and sealed using a vacuum sealing device. Finally, the meat was sterilized in a pressure steam sterilizer at 121 °C for 20 min. For the specific procedure, refer to Figure 1, taking BCP as an example and following the path indicated by the green arrow.

### 2.4. Shelf-Life Test

The shelf-life of the samples was assessed via high-temperature destructive testing [8]. The sterilized experimental and control samples were, respectively, stored in constant temperature environments at 35 °C and 45 °C; tolerance limit for storage temperature was 1 °C. Based on the data obtained from preliminary experiments, sampling and testing of the samples were conducted every 20 days commencing from the 80th day. The testing items and methods are as follows: *Salmonella* (S) was tested in accordance with GB4789.4-2024 [9]; *Listeria monocytogenes* (LM) was detected by employing the first method of GB4789.30-2016 [10]; *Staphylococcus aureus* (SA) was tested using the second method of GB4789.10-2016 [11]; Total bacterial count (TC) was measured according to GB4789.2-2022 [12]; Total coliforms value (TCV) were detected using the second method of GB4789.3-2016 [13]. According to the following formula, the shelf-life of the product under different temperature conditions can be derived [14,15].

$$Q_{10}=\frac{F_{1}}{F_{2}}\times \frac{10}{T_{2}-T_{1}}$$

$$F=\frac{T_{1}-T}{10}\times Q_{10}\times F_{1}$$

*F*_1_ is the shelf-life at *T*_1_ (35 °C), *F*_2_ is the shelf-life at *T*_2_ (45 °C), and *F* is the shelf-life at room temperature *T* (25 °C).

### 2.5. Color Difference Test

Drawing upon Wang’s research [16] methodology, the experimental and control groups underwent a cross-sectional incision (with an approximate area of 25 square centimeters and a thickness spanning from 2 to 3 cm). A color difference meter was employed to measure the surface color of the samples. The lightness (L*), red-green chromaticity (a*), yellow-blue chromaticity (b*), chroma (c*), and hue angle (*h*°) of the samples were documented. Each group of samples was measured three times repeatedly, and the mean values were computed. The chromaticity coordinates, including c* and *h*°, were calculated from the a* and b* values according to the CIE recommendations, using the following equations:

$$C*=\sqrt{{a*}^{2}+{b*}^{2}}$$

h°=atan2(b*,a*)

### 2.6. Texture Property Test

In accordance with the approach of Luzardo et al. [17], this study employed a CTX texture analyzer to conduct measurements on samples from both the experimental and control groups. The specific experimental procedures were as follows: prior to measurement, the samples were stored in an environment at 4 °C for one hour. Subsequently, filter paper was utilized to eliminate surface moisture from the samples, and they were manually sectioned into cubes with dimensions of 1 cm × 1 cm × 1 cm. All muscle samples were cut along the fiber direction. A P/40 cylinder probe was chosen for the testing process. During the test, the samples were vertically compressed along the muscle fibers to 50% of their initial height. The instrument parameters were configured, with the probe speed set at 5 mm/s before the test, 2 mm/s during the test, and 2 mm/s after the test and trigger force was 5 g. Each group of samples was measured three times, and the final result was determined as the average of these measurements.

### 2.7. Moisture Content Test

The determination was carried out using the direct drying method stipulated in GB5009.3-2016 [18] “Determination of Water in Food”.

### 2.8. Yield Test

The yield (Y) of the final meat product, representing the mass ratio of the final product to the pre-processed (cut and cleaned) meat pieces, is calculated using: $Y=\frac{m_{2}}{m_{1}}\times 100\%$ [19].

*Y*: Yield of the final meat product (%);

*m*_1_: Mass of the meat pieces after cutting and cleaning (unit: g);

*m*_2_: Mass of the final meat product (unit: g).

### 2.9. Protein Content

The protein content in meat after 200 days was determined by Kjeldahl nitrogen determination method specified in GB5009.5-2016 [20].

### 2.10. Determination of Volatile Flavor Compounds

This research utilized HS-SPME-GC-MS to examine the disparities in volatile flavor compounds between the experimental group and the control group at both the initial stage and after 200 days of long-term storage. During the experimental process, the extraction head was aged at 250 °C for 5 min. Two grams of meat specimens were minced and placed into a headspace extraction vial, followed by the addition of 3 mL of saturated NaCl solution (GC Grade). The specimens were heated and agitated at 60 °C for 20 min, then subjected to extraction for 40 min, desorbed at 250 °C for 5 min, and analyzed using the pulse non-split mode. A DB-Wax column (60 mm × 0.25 mm × 0.25 μm) was employed, with nitrogen gas (purity ≥ 99.999%) serving as the carrier gas. The flow rate was set at 1.00 mL/min, the column head pressure was maintained at 2.4 kPa, and the detector temperature was set at 250 °C. The column was initially heated to 35 °C and held for 1 min, then the temperature was elevated to 100 °C at a rate of 2 °C/min and held for 1 min; it was further increased to 170 °C at a rate of 2 °C/min and held for 1 min; finally, it was raised to 250 °C at a rate of 5 °C/min and held for 3 min. The mass spectrometry analysis conditions encompassed an electron impact ion source with an electron energy of 70 eV, both the transfer line temperature and the ion source temperature set at 250 °C, a mass scan range of *m*/*z* 30–550, full-scan mode, and standard tuning files [21]. Gas chromatography-mass spectrometry (GC-MS) can be utilized to conduct qualitative analysis and relative quantitative analysis (based on ion fragments) of volatile flavor components in meat specimens.

### 2.11. Sensory Evaluation

Sensory evaluation of the samples was performed with reference to the sensory identification requirements stipulated in the Chinese National Recommended Standard GB/T 22210-2008 [22] (Specifications for Sensory Evaluation of Meat and Meat Products), A trained panel of ten assessors (five males and five females, aged 25–35 years) was selected and underwent four weeks of training prior to the formal evaluation. All samples were labeled with random three-digit codes and presented to the assessors following a Latin square design to balance the serving order across the panel. This ensured that each sample appeared an equal number of times at each serving position. Evaluations were conducted in individual sensory booths under controlled environmental conditions: temperature of 22 ± 2 °C, relative humidity of 50 ± 5%, illumination of 500–750 lux, and background noise maintained below 40 dB. A 5 min interval was enforced between samples, during which assessors were required to rinse their mouths with distilled water to minimize carryover effects. Samples were uniformly prepared as 2 cm^3^ cubes, equilibrated to room temperature (approximately 25 °C) before serving. The sensory attributes were assessed using a 20-point intensity scale (0 = extremely poor, 20 = extremely good) for the following parameters: color (uniformity/brightness), flavor (absence of off-flavors), chewiness (mouthfeel acceptability), and texture (absence of cracks). Overall acceptability was also scored on the same 20-point scale, making the theoretical maximum total score 100 points.

### 2.12. Statistical Analysis

In this study’s statistical analysis, the paired comparison chart method in OriginPro2021 was employed, with significance marked by ‘top letters’ indicating statistically significant differences between groups. Error bars were plotted using ‘standard deviation (SD)’ to reflect data dispersion. The Dunn–Sidak method was employed for mean comparison, which provides a visual and quantitative basis for assessing inter-group differences in research conclusions by performing a corrected test on the means of multiple datasets.

## 3. Results and Discussion

### 3.1. Shelf-Life Analysis

#### 3.1.1. Total Bacterial Colony Analysis

The TC was determined according to the Chinese National Standard GB 4789.2-2022 [23], the TC in food shall not exceed 1.0 × 10^5^ CFU/g (CFU: Colony-Forming Units). Meat products that exceed this standard are regarded as non-compliant. As presented in Table 1, under the condition of 45 °C, the total bacterial counts of BS, BCP, PS, PCP, CS, and CCP all conformed to the national standard of less than 1.0 × 10^5^ CFU/g by the 140th day. Nevertheless, by the 160th day, these counts no longer met the national limit. Conversely, under the condition of 35 °C, the total bacterial counts of BS, BCP, PS, PCP, CS, and CCP remained below 1.0 × 10^5^ CFU/g throughout the 200-day storage period, satisfying the national limit requirements.

#### 3.1.2. Analysis of Coliform Bacteria

Coliforms in the samples were quantified following the method described in the Chinese National Food Safety Standard GB 4789.3-2016 [13], which specifies that the maximum allowable content of coliforms in food shall not exceed 1.0 × 10^2^ CFU/g. When this standard is exceeded, the meat products are deemed to be of non-compliant quality. As presented in Table 1, under storage conditions of 45 °C and 35 °C, the coliform content of samples from groups BS, BCP, PS, PCP, CS, and CCP all conformed to the national limit standards by the 200th day of storage, with the content being below 1.0 × 10^2^ CFU/g.

#### 3.1.3. Pathogenic Bacteria Analysis

Based on the data presented in Table 1, under the storage conditions of 45 °C and 35 °C, the contents of *Listeria monocytogenes*, *Salmonella*, and *Staphylococcus aureus* in the BS, BCP, PS, PCP, CS, and CCP groups throughout the 200 day storage period all complied with the relevant provisions of GB 29921-2016 [24] “National Food Safety Standard-Maximum Levels of Pathogenic Bacteria in Prepackaged Foods”.

#### 3.1.4. Calculate Shelf-Life

Based on the aforementioned analysis, the shelf-life of the three meat samples and their corresponding controls under a temperature of 45 °C is 140 days, whereas under a temperature of 35 °C, it surpasses 200 days (designated as 200 days). Computational results indicate that the Q10 value is approximately 1.43. In accordance with relevant formulas, the shelf-life can exceed 286 days, the propagation uncertainty for the room temperature life is 46 days (Table 2).

### 3.2. Color Difference Analysis

**Figure 2:**
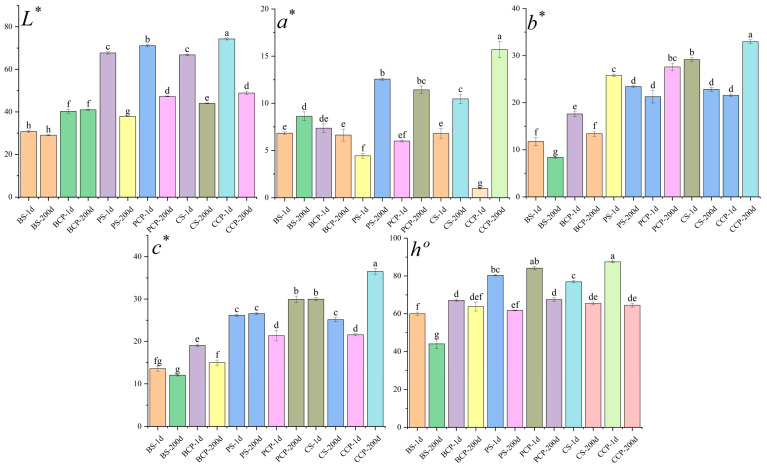
Color difference analysis; significant differences are shown by different letters in each column (*n* = 3, *p* < 0.01), 200 d (200th day), 1 d (Day 1).

As presented in Figure 2, following a 200 day experimental duration, the L* values (indicators of brightness) of both the experimental groups (BS-200, PS-200, CS-200) and the control group (BCP-200, PCP-200, CCP-200) demonstrated a downward tendency, signifying a shift towards darker colors. In the majority of samples, the a* values, which serve as indicators of the red-green hue, increased over time, suggesting a proclivity towards reddish tones; nevertheless, the a* values of BCP samples marginally decreased. Further examination disclosed that the samples in the experimental group generally manifested a tendency to shift towards reddish and bluish tones (an increase in a* values and a decrease in b* values), whereas the samples in the control group generally displayed a tendency to shift towards reddish and yellow tones (an increase in both a* and b* values). Notably, the BCP samples (a part of the control group) exhibited distinctive characteristics, with both their a* and b* values decreasing. Specifically, this phenomenon may be ascribed to the coarse attributes of beef muscle fibers. When muscle fibers are severed, their cross-section presents a regular arrangement of convex and concave structures, which are termed gratings in the field of optics. The grating effect leads to the mutual interference of incident light [25].

### 3.3. Texture Analysis

**Figure 3:**
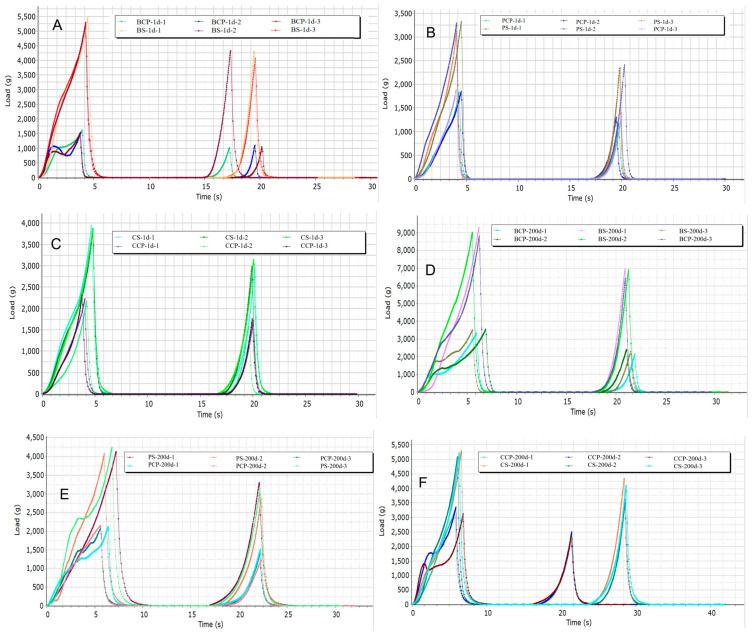
(**A**) Texture curves of BS and BCP (1 d), (**B**) texture curves of PS and PCP (1 d), (**C**) texture curves of CS and CCP (1 d), (**D**) texture curves of BS and BCP (200 d), (**E**) texture curves of PS and PCP (200 d), (**F**) texture curves of CS and CCP (200 d).

**Figure 4:**
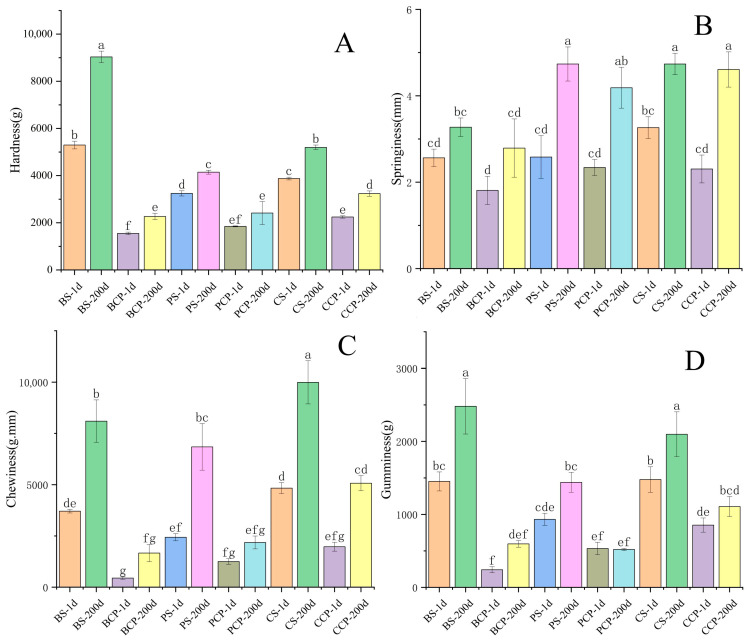
Texture analysis; significant differences are shown by different letters in each column (n = 3, *p* < 0.05), 200 d (200th day), 1 d (Day 1), (**A**) (Hardness), (**B**) (Springiness), (**C**) (Chewiness), (**D**) (Gumminess).

In this study, as depicted in Figure 3 and Figure 4, the hardness of the experimental groups (comprising BS, PS, CS) and the control group (including BCP, PCP, CCP) exhibited a significant increase over time (ranging from 1 to 200 days). By the 200-day mark, BS attained its peak hardness value of 9034, succeeded by CS with a hardness value of 5198 and PS with a value of 4145. Generally, the hardness of the experimental groups was two to four times greater than that of the control group. Regarding elasticity, both the experimental and control groups demonstrated a notable increase over time. Particularly in PS and CS, the increase in elasticity was most prominent. By the 200 day period, PS and CS reached their maximum elasticity values of 4.73, with BS closely trailing at 3.27. The disparity in elasticity between the experimental and control groups was relatively minor. In terms of chewiness, both groups showed a significant upward trend over time, with CS displaying the most substantial increase (from 4833 to 9988). By the 200 day time point, CS had the highest chewiness at 9988, followed by BS at 8100 and PS at 6843. The chewiness of the experimental group was three to five times higher than that of the control group. Except for PCP, the adhesive properties of both the experimental and control groups augmented over time. BS exhibited the highest adhesive properties at 2481, followed by CS at 2098 and PS at 1439. The adhesive properties of the experimental group were two to four times higher than those of the control group. On the first day and after 200 days, the increases in hardness and chewing strength were predominantly propelled by Maillard cross-linking and gel shrinkage. The increase in adhesion was correlated with anaerobic bacterial extracellular polysaccharides, while alterations in elasticity were a consequence of the equilibrium between oxidative inhibition and non-oxidative degradation [26]. Simultaneously, in comparison with the control group, the experimental group exhibited higher levels of hardness, elasticity, chewing performance, and adhesion. This phenomenon can be attributed to the omission of the brine-cooking process in the experimental processing method [27].

### 3.4. Moisture Content Analysis

**Figure 5:**
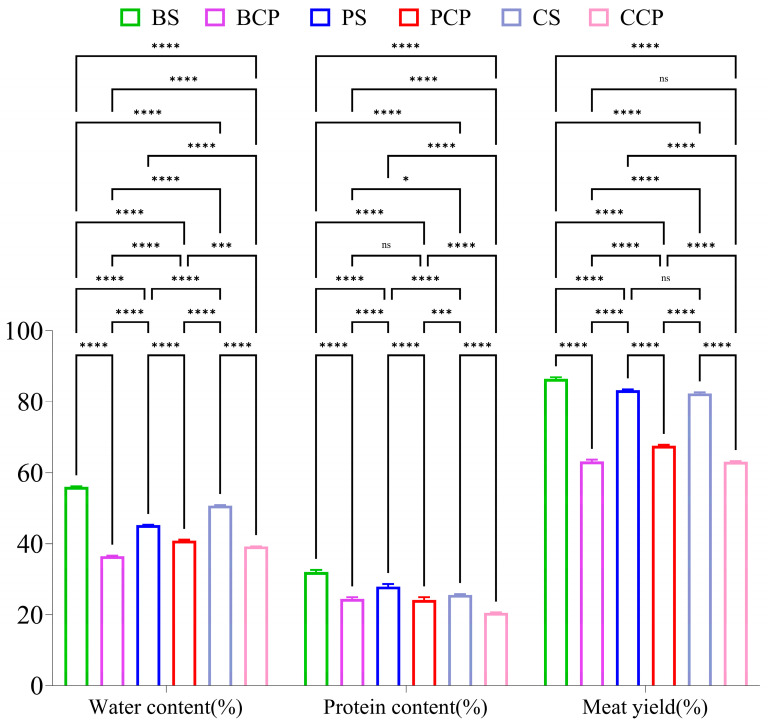
Effects of different processing methods on meat products (200 d): Effect of water content, effect of protein content, effect of yield (*n* = 3, * *p* ≤ 0.05, *** *p* < 0.001, **** *p* < 0.0001, and ns indicates not significant (*p* > 0.05).

In this study (as presented in Figure 5), the moisture content of the experimental groups (BS, PS, CS) fell within the range of 45.22% to 50.70%, whereas that of the control groups (BCP, PCP, CCP) was between 36.46% and 40.85%. The average moisture content of the experimental groups attained 48.20%, approximately 10 percentage points higher than the 38.86% in the control group. Remarkably, the BS group exhibited a notably significant difference, with its moisture content surpassing that of the BCP group by 12.23%. This disparity mainly originates from two fundamental distinctions in the processing methods: firstly, the experimental group employed direct packaging of raw meat, which precluded water loss during the brine cooking process. Secondly, the spices in the experimental group retained continuous activity, as the proteases they contained continuously decomposed collagen proteins, facilitating the formation of water-retaining gels via polysaccharide compounds [28]. These synergistic effects conferred upon the experimental products remarkable water retention capabilities.

### 3.5. Analysis of Meat Yield

As depicted in Figure 5, when compared with the control group (BCP, PCP, CCP), the yields of BS, PS, and CS were significantly elevated. Among them, BS exhibited the optimal yield, being 23.72% higher than that of BCP; CS was 19.17% higher than CCP; and PS was 15.8% higher than PCP. The experimental group employed a novel process involving vacuum packaging and sterilization of raw meat, thereby eliminating the traditional brining procedure. This approach effectively mitigated moisture evaporation and the loss of soluble nutrients (such as water-soluble vitamins and minerals) during the processing [29]. In contrast, the control group necessitated dual heating procedures, specifically brining followed by sterilization. Extended heating led to excessive moisture evaporation, nutrient depletion, over-contraction of muscle fibers, and ultimately, a lower meat yield. From the viewpoint of protein denaturation, the single-phase heating in the experimental group gave rise to more uniform protein denaturation, thereby forming a more complete gel network structure with improved water-holding capacity [30]. In the control group, repeated heating, conversely, resulted in excessive protein denaturation and a reduction in water-holding capacity [27].

### 3.6. Protein Content Analysis

As depicted in Figure 5, the protein content in the experimental groups (BS, PS, CS) was notably higher than that in the control group (BCP, PCP, CCP). Specifically, the protein content in BS was 7.57% higher than that in BCP. Likewise, the protein content in PS was 3.75% higher than that in PCP, and the protein content in CS was 5.09% higher than that in CCP. BS exhibited the highest protein content, reaching 32.03%, whereas CCP had the lowest protein content, at 20.51%. The primary cause can be attributed to the traditional brining method adopted in the control group, which necessitates a longer heating duration and results in a higher degree of protein denaturation. In contrast, the experimental group utilized direct sealing followed by high-temperature sterilization, significantly curtailing the overall heat treatment time and consequently reducing protein thermal denaturation [31]. Moreover, during the entire storage process, the experimental group ensured direct contact between spices and meat. The antioxidant constituents in spices might have decelerated the oxidative degradation of proteins. As research suggests, the polyphenolic compounds present in specific spices (e.g., Sichuan pepper and star anise) exhibit protein-protective characteristics. Additionally, the vacuum-sealing and heating method employed by the experimental group minimized oxygen exposure, thereby further mitigating protein oxidation [32]. In comparison, the control group’s exposure to air during brining increased the risk of protein oxidation.

### 3.7. Sensory Evaluation Analysis

**Figure 6:**
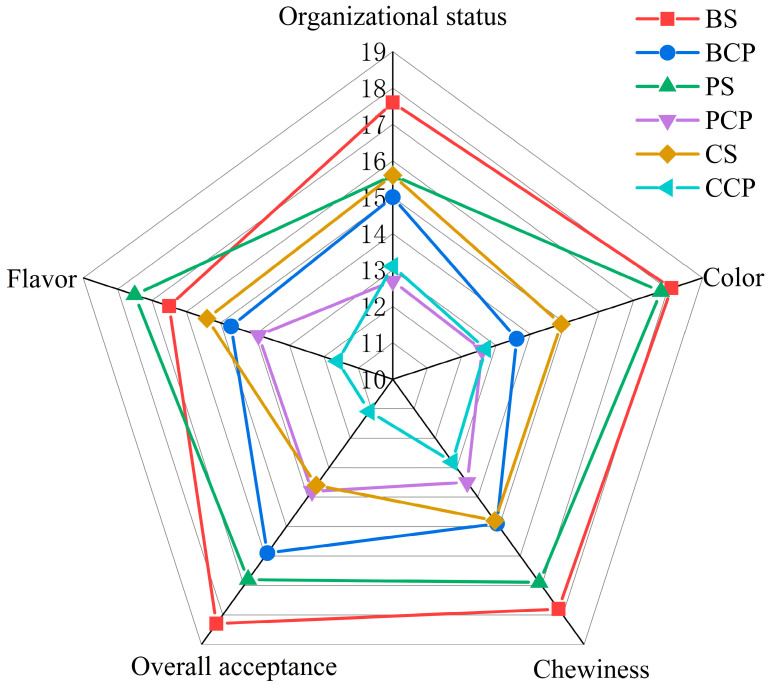
Effects of different processing methods on sensory evaluation of meat products (200 days).

As depicted in Figure 6, the experimental group exhibited remarkable superiority over the control group in all dimensions of sensory evaluation, especially in terms of color. Specifically, BS performed optimally across all evaluation indicators, attaining a comprehensive score of 88.3, which was 14.2 points higher than BCP’s score of 74.1. Simultaneously, PS scored 18.1 points higher than PCP, and CS scored 13 points higher than the CCP sample.

### 3.8. Analysis of Volatile Flavor Substances

**Figure 7:**
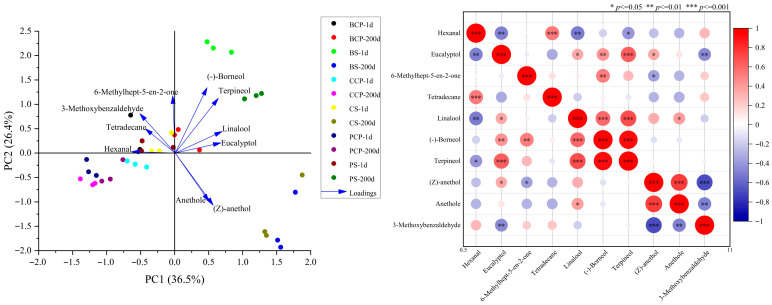
Principal component analysis and cluster analysis, 200 d (200th day), 1 d (Day 1).

This research utilized HS-SPME-GC-MS to analyze volatile flavor compounds at the initial and final stages of the shelf-life for the experimental groups (BS, PS, CS) and the control groups (BCP, PCP, CCP). As presented in Table 3, a cumulative total of 186 volatile flavor compounds were detected in all samples, encompassing acids (10), alcohols (40), aldehydes (13), alkanes (25), alkenes (24), aromatic hydrocarbons (5), esters (23), ethers (6), ketones (21), phenols (6), pyrazines (3), sulfides (2) and others (9). The experimental group exhibited a superior capacity for flavor retention compared to the control group, with a greater number of detected compounds. Key flavor components, such as alcohols, ketones, and ethers, demonstrated higher stability. Alcohols (e.g., 1-octenyl-3-alcohol) contribute to the complex flavors characteristic of mushrooms, whereas ketones (e.g., methyl geranol) impart fruity and green notes. The experimental group adopted shorter heat treatment durations and vacuum packaging to suppress oxidation, facilitating the dynamic development of flavors. Its core advantages lie in the reduction in heating time, the sustained release of spices, and the effective inhibition of oxidation, ultimately leading to the formation of more complex flavor profiles and an enhancement of storage stability. This method is more effective in preserving key flavor compounds such as alcohols, ethers, and ketones. Notably, the levels of aldehydes in the experimental group were lower than those in the corresponding control groups, suggesting more pronounced Maillard reactions and non-enzymatic browning in the control group [33]. Furthermore, the elevation of aldehydes (e.g., n-hexanal) may facilitate lipid oxidation and give rise to “aging flavors” [34]. The experimental group exhibited an absence of sulfides, whereas the control group yielded a positive result for sulfides on the initial day. It is well-established that sulfides can expedite protein degradation [35]. Based on the PCA (Principal Component Analysis) plots (Figure 7), it can be observed that under short-term (1 day) conditions, the experimental group already exhibits significant overall compositional differences compared with the control group, with the separation of BS-1 d from the control group being particularly notable. Under long-term (200 days) conditions, the overall differences between the experimental group and the control group further increase; for instance, the separation of BS-200 d and PS-200 d from the control group becomes even more pronounced. From the analysis of the heatmap of volatile compounds (Figure 7), during the short-term (1 day) period, there are extremely significant or highly significant differences in the contents of various substances such as Hexanal and Eucalyptol between the experimental and control groups. After the long-term (200 days), substances such as Benzene, 1-methoxy-4-(1-propenyl)-, (Z)-, and others still show highly significant content differences between the two groups, and the differential patterns of some substances either remain stable or undergo further changes over time. In summary, both in the short-term and long-term, there are significant differences between the experimental and control groups in terms of overall composition as well as the relative contents of specific volatile substances, with some differences showing an increasing trend over time. The results illustrate that the experimental group presented more excellent flavor characteristics in comparison to the control group. These findings suggest that innovative processing technologies possess significant potential in the food industry, facilitating the production of high-quality products with more long-lasting and stable flavors.

## 4. Conclusions

This research has devised an innovative processing methodology that eschews traditional brine curing and integrates vacuum packaging with high-temperature sterilization. This approach notably prolongs the shelf life of prepacked braised meat products while conserving their original flavor, nutritional value, and textural properties. Experimental findings indicated that both groups manifested increasing trends in hardness, elasticity, chewiness, and viscosity over time, with the experimental group demonstrating superior performance. The experimental group also exhibited higher water content, product yield, and protein content. Sensory evaluation confirmed its overall advantage in all key attributes. Analysis of volatile flavor compounds revealed that the experimental group retained flavor components more effectively, maintained stable key flavor substances, and delivered a better flavor profile. In conclusion, this approach enhanced prepacked braised meat products across multiple dimensions and showed potential for food industry applications. In the following research, alternative packaging materials and sterilization parameters need to be investigated to further improve prepacked braised meat products quality and shelf life.

## Figures and Tables

**Table 1:** Shelf-life microbial analysis.

Sample	Temperature/℃	TCV/(CFU/g)	TC/(CFU/g)	S/(CFU/25 g)	SA/(CFU/g)	LM/(CFU/g)
80 d						
BS	35	<10	<10	-	<10	-
	45	<10	<10	-	<10	-
BCP	35	<10	<10	-	<10	-
	45	<10	<10	-	<10	-
PS	35	<10	<10	-	<10	-
	45	<10	<10	-	<10	-
PCP	35	<10	<10	-	<10	-
	45	<10	<10	-	<10	-
CS	35	<10	<10	-	<10	-
	45	<10	<10	-	<10	-
CCP	35	<10	<10	-	<10	-
	45	<10	<10	-	<10	-
100 d						
BS	35	<10	<10	-	<10	-
	45	<10	<10	-	<10	-
BCP	35	<10	<10	-	<10	-
	45	<10	<10	-	<10	-
PS	35	<10	<10	-	<10	-
	45	<10	<10	-	<10	-
PCP	35	<10	<10	-	<10	-
	45	<10	<10	-	<10	-
CS	35	<10	<10	-	<10	-
	45	<10	<10	-	<10	-
CCP	35	<10	<10	-	<10	-
	45	<10	<10	-	<10	-
120 d						
BS	35	<10	<10	-	<10	-
	45	<10	<10	-	<10	-
BCP	35	<10	<10	-	<10	-
	45	<10	<10	-	<10	-
PS	35	<10	<10	-	<10	-
	45	<10	<10	-	<10	-
PCP	35	<10	<10	-	<10	-
	45	<10	<10	-	<10	-
CS	35	<10	<10	-	<10	-
	45	<10	<10	-	<10	-
CCP	35	<10	<10	-	<10	-
	45	<10	<10	-	<10	-
140 d						
BS	35	<10	<10	-	<10	-
	45	15	<10	-	<10	-
BCP	35	<10	<10	-	<10	-
	45	130	<10	-	<10	-
PS	35	<10	<10	-	<10	-
	45	44	<10	-	<10	-
PCP	35	<10	<10	-	<10	-
	45	93	<10	-	<10	-
CS	35	<10	<10	-	<10	-
	45	65	<10	-	<10	-
CCP	35	<10	<10	-	<10	-
	45	110	<10	-	<10	-
160 d						
BS	35	<10	<10	-	<10	-
	45	>1 × 10^5^	<10	-	<10	-
BCP	35	<10	<10	-	<10	-
	45	>1 × 10^5^	<10	-	<10	-
PS	35	<10	<10	-	<10	-
	45	>1 × 10^5^	<10	-	<10	-
PCP	35	<10	<10	-	<10	-
	45	>1 × 10^5^	<10	-	<10	-
CS	35	<10	<10	-	<10	-
	45	>1 × 10^5^	<10	-	<10	-
CCP	35	<10	<10	-	<10	-
	45	>1 × 10^5^	<10	-	<10	-
180 d						
BS	35	<10	<10	-	<10	-
BCP	35	<10	<10	-	<10	-
PS	35	<10	<10	-	<10	-
PCP	35	<10	<10	-	<10	-
CS	35	<10	<10	-	<10	-
CCP	35	<10	<10	-	<10	-
200 d						
BS	35	<10	<10	-	<10	-
BCP	35	<10	<10	-	<10	-
PS	35	<10	<10	-	<10	-
PCP	35	<10	<10	-	<10	-
CS	35	<10	<10	-	<10	-
CCP	35	<10	<10	-	<10	-

*Salmonella* (S), *Listeria monocytogenes* (LM), *Staphylococcus aureus* (SA), Total bacterial count (TC), Total coliforms value (TCV), “-” not detected.

**Table 2:** The propagated uncertainty for the extrapolated room-temperature life.

Parameter	Time	Uncertainty (δ)	Relative Uncertainty
*F*_2_	140 days	±20 days	14.30%
*F*_1_	200 days	±20 days	10.00%
*Q*_10_	1.43	±0.25	17.40%
*F*	286 days	±46 days	16.20%

**Table 3:** Volatile flavor substances.

NO.	Compounds	CAS	Similarity	Quantitative/ Reference Ions	Category	BS-1	BCP-1	BS-200	BCP-200	PS-1	PCP-1	PS-200	PCP-2	CS-1	CCP-1	CS-200	CCP-200
1	Dimethyl disulfide	624-92-0	99	94, 79, 45	Ethers	-	-	-	-	-	5.564 ± 0.585	-	8.204 ± 0.879	-	-	-	-
2	Hexanal	66-25-1	99	56, 44, 41	Aldehydes	0.991 ± 0.031	1.698 ± 0.098	0.022 ± 0.004	0.917 ± 0.065	0.295 ± 0.078	1.240 ± 0.086	0.049 ± 0.001	2.737 ± 0.206	0.343 ± 0.041	0.633 ± 0.028	0.115 ± 0.029	2.081 ± 0.136
3	Dodecane	112-40-3	91	57, 43, 71	Alkanes	0.486 ± 0.206	-	0.023 ± 0.004	0.374 ± 0.136	0.378 ± 0.225	0.372 ± 0.335	-	0.388 ± 0.252	0.158 ± 0.022	0.239 ± 0.087	-	0.519 ± 0.173
4	Eucalyptol	470-82-6	98	43, 108, 81	Alcohols	3.286 ± 1.522	1.636 ± 0.644	2.010 ± 0.498	1.671 ± 0.948	1.999 ± 1.125	0.609 ± 0.313	5.245 ± 0.293	1.317 ± 0.548	1.921 ± 0.786	0.551 ± 0.355	6.436 ± 1.290	0.516 ± 0.348
5	2-Pentyl-furan,	3777-69-3	97	81, 82, 138	Others	-	-	-	1.518 ± 0.812	-	-	-	1.880 ± 0.627	-	1.209 ± 0.718	-	5.848 ± 2.148
6	4-Octanone	589-63-9	96	71, 57, 85	Ketones	0.237 ± 0.097	-	-	-	-	0.349 ± 0.154	-	0.234 ± 0.058	0.146 ± 0.066	0.196 ± 0.020	-	0.445 ± 0.040
7	Cyclohexane, ethoxy	932-92-3	98	85, 57, 41	Alkanes	-	-	-	-	-	0.823 ± 0.492	-	1.691 ± 0.372	-	-	-	-
8	3-methyl-5-propylnonane	31081-18-2	96	71, 57, 85	Alkanes	-	-	-	0.902 ± 0.448	-	-	-	0.280 ± 0.179	-	-	-	-
9	Tridecane	629-50-5	98	57, 71, 43	Alkanes	-	-	0.049 ± 0.032	0.483 ± 0.593	0.287 ± 0.394	-	0.155 ± 0.040	0.684 ± 0.423	-	-	-	-
10	2-Heptanol	543-49-7	96	45, 55, 83	Alcohols	2.341 ± 1.568	-	1.467 ± 0.036	-	-	0.448 ± 0.317	2.994 ± 0.137	0.918 ± 0.039	-	0.395 ± 0.032	0.691 ± 0.151	-
11	6-Methylhept-5-en-2-one	110-93-0	100	43, 108, 41	Ketones	10.329 ± 0.223	7.724 ± 0.374	3.748 ± 0.820	9.768 ± 0.342	6.093 ± 0.459	4.299 ± 0.305	7.093 ± 0.294	7.715 ± 0.604	11.056 ± 1.078	8.589 ± 0.177	4.194 ± 0.891	3.550 ± 0.091
12	Dimethyl trisulfide	3658-80-8	100	126, 76, 45	Ethers	12.460 ± 1.284	12.131 ± 0.361	0.107 ± 0.128	14.371 ± 3.359	-	35.478 ± 5.654	-	14.159 ± 10.207	5.700 ± 1.874	2.443 ± 0.136	0.618 ± 0.138	9.044 ± 3.260
13	2-Nonanone	821-55-6	97	58, 43, 71	Ketones	1.949 ± 0.054	-	1.019 ± 0.165	0.765 ± 0.021	6.097 ± 0.736	0.515 ± 0.018	1.807 ± 0.086	0.559 ± 0.045	14.371 ± 0.548	12.644 ± 0.190	0.589 ± 0.137	0.648 ± 0.007
14	(E)-1-methyl-4-(prop-1-en-1-yl)benzene	2077-30-7	98	132, 117, 115	Alkanes	0.515 ± 0.036	0.721 ± 0.076	0.335 ± 0.040	0.538 ± 0.030	-	-	0.587 ± 0.003	0.444 ± 0.021	0.119 ± 0.161	-	0.524 ± 0.069	-
15	Tetradecane	629-59-4	95	57, 71, 43	Alkanes	1.676 ± 0.179	1.215 ± 0.276	0.177 ± 0.135	1.668 ± 1.206	0.631 ± 0.499	0.837 ± 0.597	0.281 ± 0.061	1.161 ± 0.062	0.681 ± 0.515	0.591 ± 0.424	0.142 ± 0.032	2.272 ± 0.521
16	1-Octen-3-ol	3391-86-4	99	57, 43, 72	Alcohols	-	1.286 ± 0.094	-	6.125 ± 0.407	0.944 ± 0.081	2.479 ± 0.116	-	7.453 ± 0.477	-	1.827 ± 0.049	0.962 ± 0.211	10.899 ± 0.366
17	Benzaldehyde	100-52-7	100	106, 105, 77	Aldehydes	3.513 ± 0.103	18.347 ± 1.280	0.587 ± 0.104	7.854 ± 0.296	3.723 ± 0.264	8.033 ± 0.578	-	8.941 ± 1.237	3.037 ± 0.424	4.306 ± 0.022	0.434 ± 0.094	5.552 ± 0.132
18	Heptadecane	629-78-7	99	57, 71, 43	Alkanes	0.128 ± 0.088	0.134 ± 0.076	-	0.349 ± 0.227	-	0.500 ± 0.477	0.494 ± 0.082	1.613 ± 0.693	0.067 ± 0.032	0.097 ± 0.044	-	0.109 ± 0.132
19	Linalool	78-70-6	99	71, 73, 55	Alcohols	3.081 ± 0.032	1.006 ± 0.135	3.473 ± 0.659	1.250 ± 0.096	1.650 ± 0.097	0.527 ± 0.040	1.980 ± 0.086	0.646 ± 0.179	1.181 ± 0.137	0.692 ± 0.008	1.363 ± 0.316	0.565 ± 0.018
20	(-)-Terpinen-4-ol	20126-76-5	97	71, 111, 93	Alcohols	1.874 ± 0.039	0.894 ± 0.114	1.779 ± 0.349	1.037 ± 0.087	1.236 ± 0.059	-	-	0.765 ± 0.198	1.232 ± 0.173	-	3.092 ± 0.690	0.571 ± 0.027
21	(Z)-2-octen-1-ol	26001-58-1	95	57, 41, 55	Alcohols	-	-	-	-	-	-	-	0.250 ± 0.130	-	0.121 ± 0.009	-	0.380 ± 0.012
22	Heneicosane	629-94-7	97	57, 71, 85	Alkanes	1.180 ± 0.125	0.842 ± 0.240	-	1.310 ± 0.056	0.829 ± 0.143	1.341 ± 0.186	0.250 ± 0.070	0.457 ± 0.350	0.907 ± 0.134	0.440 ± 0.068	0.122 ± 0.013	1.469 ± 0.211
23	Estragole	140-67-0	97	148, 147, 117	Ethers	0.626 ± 0.016	0.466 ± 0.045	2.222 ± 0.378	0.511 ± 0.020	0.875 ± 0.053	-	0.511 ± 0.025	0.229 ± 0.141	0.679 ± 0.480	0.886 ± 0.008	1.305 ± 0.252	0.412 ± 0.002
24	(-)-Borneol	464-45-9	97	95, 110, 93	Alcohols	4.640 ± 0.111	2.725 ± 0.243	2.240 ± 0.446	3.023 ± 0.127	1.622 ± 0.098	1.266 ± 0.097	4.312 ± 0.160	1.998 ± 0.487	2.061 ± 0.265	1.889 ± 0.017	2.070 ± 0.457	1.625 ± 0.025
25	Terpineol	98-55-5	98	59, 93, 1 21	Alcohols	2.453 ± 0.070	1.246 ± 0.167	1.227 ± 0.257	1.454 ± 0.097	1.161 ± 0.028	0.613 ± 0.051	2.304 ± 0.053	0.599 ± 0.276	0.797 ± 0.104	0.704 ± 0.009	1.562 ± 0.354	0.605 ± 0.003
26	(Z)-anethol	25679-28-1	98	148, 147, 117	Alkenes	0.493 ± 0.034	1.066 ± 0.107	2.896 ± 0.245	1.565 ± 0.029	0.499 ± 0.058	0.459 ± 0.030	0.661 ± 0.011	1.248 ± 0.407	0.966 ± 0.020	0.867 ± 0.018	2.928 ± 0.382	1.054 ± 0.009
27	Anethole	104-46-1	100	148, 147, 117	Ethers	22.976 ± 1.001	32.954 ± 3.484	58.532 ± 6.828	31.522 ± 1.664	27.984 ± 0.897	14.154 ± 0.598	26.974 ± 0.517	30.849 ± 6.821	29.240 ± 2.134	30.008 ± 0.409	41.825 ± 11.890	28.459 ± 0.580
28	3-Methoxybenzaldehyde	591-31-1	96	135, 136, 77	Aldehydes	3.029 ± 0.240	3.725 ± 0.864	1.186 ± 0.318	1.450 ± 0.096	3.931 ± 0.116	3.143 ± 0.167	2.212 ± 0.259	2.210 ± 0.962	2.522 ± 0.295	2.604 ± 0.164	0.802 ± 0.168	2.794 ± 0.110
29	Undecane	1120-21-4	93	57, 43, 71	Alkanes	-	-	-	-	-	0.256 ± 0.032	-	-	-	0.118 ± 0.016	-	0.413 ± 0.079
30	Isopropyl Pentanoate	18362-97-5	95	85, 103, 43	Esters	0.342 ± 0.033	0.418 ± 0.026	-	0.417 ± 0.041	0.196 ± 0.026	0.519 ± 0.048	-	-	0.218 ± 0.030	0.241 ± 0.022	0.044 ± 0.006	0.489 ± 0.040
31	Dodecane,4,6-dimethyl	61141-72-8	97	71, 57, 85	Alkanes	0.066 ± 0.084	0.695 ± 0.154	-	0.153 ± 0.180	0.104 ± 0.021	0.579 ± 0.349	0.108 ± 0.011	-	-	0.232 ± 0.017	0.094 ± 0.028	0.165 ± 0.074
32	Octadecane	593-45-3	97	57, 71, 43	Alkanes	0.271 ± 0.171	-	-	-	-	1.325 ± 0.969	-	-	1.465 ± 0.042	-	-	0.322 ± 0.035
33	2,6,10-Trimethyltridecane	3891-99-4	92	71, 57, 85	Alkanes	0.575 ± 0.014	-	-	-	-	0.403 ± 0.071	0.071 ± 0.014	-	-	0.130 ± 0.014	-	-
34	Eicosane	112-95-8	96	71, 85, 57	Alkanes	0.293 ± 0.044	0.220 ± 0.111	0.137 ± 0.039	0.546 ± 0.007	0.423 ± 0.064	0.399 ± 0.031	-	-	0.139 ± 0.013	0.213 ± 0.029	-	0.228 ± 0.052
35	Nonadecane	629-92-5	99	57, 71, 43	Alkanes	2.025 ± 0.133	1.645 ± 0.431	0.308 ± 0.096	2.501 ± 0.139	-	2.514 ± 0.052	-	-	-	-	0.093 ± 0.014	-
36	1-Dodecanol	112-53-8	91	56, 57, 55	Alcohols	-	-	-	-	-	0.204 ± 0.022	-	-	-	-	-	-
37	Terpinen-4-ol	562-74-3	96	71, 93, 111	Alcohols	-	-	-	-	-	0.593 ± 0.047	2.550 ± 0.133	-	-	0.607 ± 0.012	-	-
38	Pentasulfide, dimethyl	7330-31-6	92	158, 79, 80	Sulfide	-	1.393 ± 0.214	-	-	-	2.589 ± 1.953	-	-	-	-	-	-
39	2,4-dimethylbenzaldehyde	15764-16-6	94	133, 134, 105	Aldehydes	0.476 ± 0.019	-	-	-	-	0.570 ± 0.101	0.182 ± 0.007	-	0.360 ± 0.063	0.562 ± 0.011	0.095 ± 0.021	0.635 ± 0.034
40	2,2,4-Trimethyl-1,3-pentanediol diisobutyrate	6846-50-0	92	71, 43, 159	Esters	-	-	0.109 ± 0.039	-	1.440 ± 0.240	0.727 ± 0.073	-	-	0.932 ± 0.060	1.119 ± 0.155	0.112 ± 0.024	1.235 ± 0.103
41	Hexadecanal	629-80-1	97	82, 57, 43	Aldehydes	1.081 ± 0.097	0.292 ± 0.070	-	-	-	0.818 ± 0.084	-	-	-	-	-	-
42	AmphetOthers	300-62-9	91	44, 91, 92	Others	-	-	-	0.521 ± 0.083	-	-	-	-	-	-	-	-
43	3,3,5-trimethylheptane	7154-80-5	96	71, 57, 43	Alkanes	-	-	-	0.243 ± 0.012	-	-	-	-	-	-	-	-
44	Carbamic acid, monoammonium salt	1111-78-0	97	44, 45, 30	Others	-	-	-	0.348 ± 0.289	-	-	-	-	-	-	-	-
45	o-Cymene	527-84-4	94	119, 134, 91	Alcohols	-	1.219 ± 0.598	0.436 ± 0.301	0.549 ± 0.508	-	-	1.005 ± 0.049	-	-	0.134 ± 0.164	2.158 ± 0.275	-
46	Dihydro Tagetone	1879-00-1	98	85, 57, 41	Ketones	-	-	-	1.281 ± 0.579	-	-	-	-	-	-	-	-
47	2-Isopropyl-5-methyl-1-hexanol	2051-33-4	98	71, 57, 43	Alcohols	-	-	-	0.168 ± 0.012	-	-	-	-	-	-	-	-
48	Cyclooctyl alcohol	696-71-9	98	57, 41, 55	Alcohols	-	-	-	0.497 ± 0.026	-	-	-	-	-	-	-	-
49	Acetophenone	98-86-2	97	105, 77, 120	Ketones	-	-	0.146 ± 0.032	0.896 ± 0.033	0.361 ± 0.027	-	0.161 ± 0.008	-	0.540 ± 0.150	0.399 ± 0.017	-	-
50	Citronellol	106-22-9	98	69, 41, 81	Alcohols	0.901 ± 0.051	0.361 ± 0.068	0.407 ± 0.092	0.549 ± 0.035	0.101 ± 0.025	-	1.001 ± 0.039	-	-	0.314 ± 0.013	0.764 ± 0.162	-
51	2-Acetyl-1H-pyrrole	1072-83-9	96	94, 109, 66	Others	0.576 ± 0.048	-	0.505 ± 0.213	0.801 ± 0.083	1.445 ± 0.031	-	0.916 ± 0.119	-	0.388 ± 0.074	-	-	-
52	2,4-Di-tert-butylphenol	96-76-4	91	191, 206, 57	Phenols	-	0.467 ± 0.031	-	0.511 ± 0.024	0.281 ± 0.034	-	-	-	0.307 ± 0.016	0.471 ± 0.046	0.065 ± 0.013	0.611 ± 0.055
53	3-Methyl-2-Heptanone	2371-19-9	96	43, 72, 85	Ketones	-	0.846 ± 0.291	-	-	-	-	-	-	-	-	-	-
54	5,6-Decanedione	5579-73-7	99	85, 57, 41	Ketones	-	1.164 ± 0.900	-	-	-	-	-	-	-	-	-	-
55	n-Caproic acid vinyl ester	3050-69-9	98	43, 99, 71	Esters	0.797 ± 0.583	1.013 ± 0.176	-	-	-	-	-	-	-	-	-	-
56	5-Methyl-2-hexanol	627-59-8	98	45, 55, 43	Alcohols	-	0.954 ± 0.016	-	-	-	-	-	-	-	-	-	-
57	2,5-Dimethylbenzaldehyde	5779-94-2	92	133, 134, 105	Aldehydes	-	0.457 ± 0.023	-	-	-	-	-	-	-	-	-	-
58	o-Anisaldehyde	135-02-4	95	136, 77, 135	Aldehydes	-	0.162 ± 0.006	-	-	-	-	-	-	-	-	-	-
59	Cinnamic aldehyde	14371-10-9	99	131, 132, 103	Aldehydes	0.288 ± 0.028	2.392 ± 0.443	0.230 ± 0.068	-	0.595 ± 0.046	-	-	-	-	0.250 ± 0.024	0.151 ± 0.030	-
60	Coumarin	91-64-5	93	146, 118, 90	Ketones	-	0.449 ± 0.137	-	-	-	-	-	-	-	-	-	-
61	Alpha-pinene	80-56-8	99	93, 91, 92	Alkenes	-	-	-	-	-	-	0.348 ± 0.021	-	-	-	-	-
62	Toluene	108-88-3	96	91, 92, 71	Aromatics	-	-	-	-	-	-	0.273 ± 0.065	-	0.989 ± 0.132	0.890 ± 0.102	-	-
63	Camphene	79-92-5	99	93, 121, 79	Alkenes	-	-	0.175 ± 0.023	-	-	-	0.800 ± 0.032	-	-	0.180 ± 0.023	1.317 ± 0.357	-
64	α-Phellandrene	99-83-2	98	93, 91, 92	Alkenes	0.167 ± 0.008	-	0.228 ± 0.038	-	-	-	0.509 ± 0.016	-	-	-	0.662 ± 0.144	-
65	β-Myrcene	123-35-3	98	93, 69, 41	Alkenes	-	-	-	-	-	-	0.674 ± 0.012	-	-	-	-	-
66	alpha-Terpinene	99-86-5	97	121, 93, 136	Alkenes	-	-	0.135 ± 0.025	-	-	-	0.202 ± 0.008	-	-	-	0.360 ± 0.035	-
67	β-phellandrene	555-10-2	95	93, 91, 136	Alkenes	-	-	0.642 ± 0.257	-	-	-	2.092 ± 0.245	-	-	-	7.080 ± 1.824	-
68	2-Heptanone	110-43-0	98	43, 58, 71	Ketones	0.763 ± 0.028	-	0.421 ± 0.207	-	-	-	0.395 ± 0.224	-	-	-	0.410 ± 0.105	1.249 ± 0.736
69	trans-.beta.-Ocimene	3779-61-1	98	93, 91, 92	Alkenes	-	-	-	-	-	-	0.264 ± 0.024	-	-	-	0.559 ± 0.071	-
70	gamma.-Terpinene	99-85-4	98	93, 91, 136	Alkenes	-	-	0.234 ± 0.077	-	-	-	0.179 ± 0.126	-	-	-	0.615 ± 0.098	-
71	(Z)-3,7-dimethylocta-1,3,6,-triene	3338-55-4	99	93, 91, 80	Alkenes	-	-	0.293 ± 0.096	-	-	-	0.455 ± 0.001	-	-	-	-	-
72	Styrene	100-42-5	98	104, 103, 78	Alkenes	0.267 ± 0.060	-	0.140 ± 0.021	-	3.234 ± 1.439	-	0.219 ± 0.015	-	5.615 ± 2.131	5.928 ± 0.294	0.241 ± 0.058	-
73	Terpinolene	586-62-9	99	121, 93, 136	Alkenes	-	-	0.287 ± 0.042	-	-	-	0.251 ± 0.002	-	-	-	0.616 ± 0.106	-
74	2-Heptanol, acetate	5921-82-4	100	43, 87, 56	Esters	-	-	0.217 ± 0.021	-	-	-	0.746 ± 0.036	-	-	-	0.363 ± 0.094	-
75	1-Methyl-3-Prop-1-En-2-Ylbenzene	1124-20-5	93	132, 117, 115	Aromatics	-	-	0.041 ± 0.001	-	-	-	0.060 ± 0.001	-	-	-	-	-
76	2,3,5-Trimethylpyrazine	14667-55-1	99	122, 42, 81	Pyrazine	-	-	0.358 ± 0.007	-	-	-	0.415 ± 0.012	-	-	-	-	-
77	Acetic acid	64-19-7	98	43, 45, 60	Acids	2.681 ± 2.446	-	1.107 ± 0.748	-	3.654 ± 2.802	-	2.003 ± 0.185	-	1.431 ± 0.740	0.892 ± 0.626	-	-
78	Copaene	3856-25-5	91	105, 119, 161	Alkenes	-	-	0.122 ± 0.002	-	-	-	0.029 ± 0.008	-	-	-	0.084 ± 0.024	-
79	2-Nonanol, acetate	14936-66-4	95	43, 87, 55	Esters	-	-	-	-	-	-	0.118 ± 0.009	-	-	-	0.071 ± 0.020	-
80	(+)-2-Bornanone	464-49-3	97	95, 81, 108	Ketones	-	-	0.165 ± 0.062	-	-	-	0.208 ± 0.034	-	-	-	0.074 ± 0.015	-
81	2-Nonanol	628-99-9	94	45, 69, 55	Alcohols	-	-	-	-	-	-	1.245 ± 0.053	-	-	0.455 ± 0.006	-	-
82	(Z)-para-2-menthen-1-ol	29803-82-5	95	43, 93, 139	Alcohols	-	-	-	-	-	-	0.036 ± 0.006	-	-	-	0.022 ± 0.005	-
83	Bornyl acetate	76-49-3	99	95, 93, 121	Esters	-	-	-	-	-	-	0.330 ± 0.005	-	-	-	0.303 ± 0.064	-
84	Camphene hydrate	465-31-6	96	71, 43, 69	Alcohols	-	-	-	-	-	-	0.090 ± 0.004	-	-	-	-	-
85	2-Undecanone	112-12-9	95	58, 43, 59	Ketones	-	-	-	-	0.090 ± 0.016	-	1.039 ± 0.044	-	-	0.395 ± 0.003	-	-
86	Trans-2-Octen-1-ol	18409-17-1	96	57, 41, 55	Alcohols	-	-	-	-	-	-	0.056 ± 0.007	-	-	-	-	0.292 ± 0.004
87	Butanoic acid	107-92-6	94	60, 73, 42	Acids	-	-	-	-	0.369 ± 0.056	-	0.230 ± 0.027	-	-	-	-	-
88	6-Octen-1-ol, 3,7-dimethyl-, acetate	150-84-5	97	81, 95, 69	Esters	-	-	-	-	-	-	0.123 ± 0.011	-	-	-	0.198 ± 0.038	-
89	alpha,alpha-Dimethyl-4-Methylenecyclohexanemethanol	7299-42-5	97	59, 81, 93	Alcohols	-	-	0.083 ± 0.017	-	0.134 ± 0.005	-	0.228 ± 0.005	-	-	-	0.183 ± 0.042	-
90	4-Isopropylcyclohex-2-En-1-One	500-02-7	94	96, 95, 43	Ketones	-	-	-	-	-	-	0.057 ± 0.009	-	-	-	0.109 ± 0.028	-
91	α-Terpinyl acetate	80-26-2	100	121, 93, 136	Esters	-	-	-	-	0.446 ± 0.008	-	0.715 ± 0.032	-	0.458 ± 0.099	0.733 ± 0.031	1.051 ± 0.219	0.453 ± 0.022
92	Pentanoic acid	109-52-4	94	60, 73, 41	Acids	-	-	-	-	0.497 ± 0.122	-	0.138 ± 0.083	-	-	-	-	-
93	(1S,4R,5R)-1,3,3-Trimethyl-2-oxabicyclo[2.2.2]octan-5-yl acetate	81781-24-0	92	43, 109, 137	Esters	-	-	-	-	-	-	0.069 ± 0.004	-	-	-	-	-
94	Geranyl acetate	105-87-3	99	69, 68, 41	Esters	0.558 ± 0.083	-	0.141 ± 0.045	-	-	-	1.193 ± 0.106	-	-	-	1.242 ± 0.210	-
95	4′-Methylacetophenone	122-00-9	93	119, 91, 134	Ketones	-	-	-	-	-	-	0.095 ± 0.005	-	-	-	-	-
96	Benzenecarboximidic acid, N-hydroxy-, methyl ester_	67160-14-9	99	133, 151, 135	Others	-	-	-	-	15.486 ± 1.737	-	5.949 ± 0.839	-	-	-	-	-
97	cis-3,7-Dimethyl-2,6-octadien-1-ol	106-25-2	90	69, 41, 93	Alcohols	-	-	0.064 ± 0.016	-	-	-	0.081 ± 0.011	-	-	-	0.123 ± 0.031	-
98	1-(2-Butoxyethoxy)-Ethanol	54446-78-5	97	57, 45, 41	Alcohols	-	-	-	-	-	-	0.177 ± 0.053	-	-	-	-	-
99	Hexanoic acid	142-62-1	99	60, 73, 41	Acids	-	-	-	-	0.738 ± 0.008	-	0.528 ± 0.144	-	-	-	-	-
100	Geraniol	106-24-1	100	69, 41, 68	Alcohols	3.920 ± 0.350	-	1.460 ± 0.479	-	-	-	2.842 ± 0.247	-	-	0.538 ± 0.030	2.838 ± 0.662	-
101	2-(2-Butoxyethoxy)ethyl acetate	124-17-4	94	87, 57, 43	Esters	-	-	-	-	-	-	0.096 ± 0.019	-	-	-	-	-
102	Methyleugenol	93-15-2	100	178, 163, 147	Phenols	-	-	0.401 ± 0.124	-	-	-	0.391 ± 0.039	-	0.145 ± 0.017	0.179 ± 0.014	0.253 ± 0.055	-
103	Octanoic acid	124-07-2	98	60, 73, 43	Acids	-	-	-	-	0.240 ± 0.168	-	0.223 ± 0.054	-	-	-	-	-
104	(1S,2S,4R)-(-)-alpha, alpha-Dimethyl-1-Vinyl-o-Menth-8-Ene-4-Methanol	639-99-6	93	73, 59, 107	Alcohols	-	-	-	-	-	-	0.037 ± 0.004	-	-	-	0.020 ± 0.005	-
105	Benzoic acid, 4-methoxy-, methyl ester	121-98-2	95	135, 166, 77	Esters	-	-	0.137 ± 0.049	-	-	-	0.230 ± 0.029	-	-	-	0.098 ± 0.022	-
106	Ethyl (E)-cinnamate; Ethyl trans-cinnamate; trans-3-Phenyl-2-propenoic acid ethyl ester	4192-77-2	97	131, 103, 176	Esters	-	-	-	-	-	-	4.062 ± 0.340	-	0.947 ± 0.135	-	-	-
107	Nonanoic acid	112-05-0	98	73, 60, 57	Acids	-	-	-	-	0.514 ± 0.174	-	0.194 ± 0.032	-	-	0.239 ± 0.095	-	-
108	3-Allyl-6-methoxyphenol	501-19-9	99	164, 149, 131	Phenols	-	-	0.161 ± 0.077	-	0.385 ± 0.014	-	0.475 ± 0.056	-	-	0.222 ± 0.017	0.129 ± 0.037	0.258 ± 0.008
109	4-Methoxyphenylacetone	122-84-9	98	121, 164, 122	Ketones	-	-	0.418 ± 0.140	-	0.727 ± 0.021	-	0.744 ± 0.097	-	0.467 ± 0.046	0.612 ± 0.056	0.328 ± 0.064	-
110	(E)-1,2-Dimethoxy-4-(1-propenyl)benzene	6379-72-2	96	178, 163, 107	Phenols	-	-	-	-	-	-	0.169 ± 0.011	-	-	-	0.180 ± 0.038	-
111	n-Decanoic acid	334-48-5	92	73, 60, 129	Acids	-	-	-	-	-	-	0.065 ± 0.012	-	-	-	-	-
112	3-Phenyl-2-propene-1-ol	104-54-1	98	92, 91, 134	Alcohols	0.159 ± 0.027	-	0.318 ± 0.162	-	0.189 ± 0.009	-	0.282 ± 0.031	-	-	-	0.114 ± 0.030	-
113	Eugenol	97-53-0	99	164, 149, 131	Phenols	-	-	-	-	-	-	0.286 ± 0.024	-	-	-	-	-
114	2-Hydroxycinnamic acid	614-60-8	98	146, 118, 90	Acids	0.438 ± 0.053	-	0.387 ± 0.181	-	0.740 ± 0.042	-	0.561 ± 0.078	-	0.379 ± 0.061	-	0.120 ± 0.025	-
115	Ethyl p-methoxycinnamate	1929-30-2	100	161, 206, 134	Esters	-	-	-	-	-	-	1.580 ± 0.139	-	-	0.833 ± 0.091	0.351 ± 0.062	1.052 ± 0.100
116	Ethylbenzene	100-41-4	95	91, 106, 78	Aromatics	-	-	-	-	0.254 ± 0.029	-	-	-	0.547 ± 0.052	0.450 ± 0.025	-	-
117	p-Xylene	106-42-3	95	91, 106, 105	Alcohols	-	-	-	-	0.254 ± 0.022	-	-	-	0.517 ± 0.034	0.402 ± 0.021	-	-
118	o-Xylene	95-47-6	96	91, 106, 105	Aromatics	-	-	-	-	0.458 ± 0.043	-	-	-	0.995 ± 0.060	0.792 ± 0.057	-	-
119	Hexanoic acid, ethyl ester	123-66-0	98	88, 99, 43	Esters	-	-	-	-	0.710 ± 0.460	-	-	-	1.188 ± 0.458	1.837 ± 0.071	-	-
120	2-Methyldodecane	1560-97-0	97	57, 43, 85	Alkanes	-	-	-	-	0.046 ± 0.027	-	-	-	-	-	-	-
121	Hydroxyacetone	116-09-6	98	43, 31, 74	Ketones	-	-	-	-	0.395 ± 0.551	-	-	-	-	-	-	-
122	1-Hexanol	111-27-3	92	56, 43, 55	Alcohols	-	-	-	-	0.064 ± 0.033	-	-	-	0.264 ± 0.022	0.378 ± 0.002	0.217 ± 0.048	0.400 ± 0.023
123	Nonanal	124-19-6	96	57, 41, 56	Aldehydes	-	-	-	-	0.165 ± 0.056	-	-	-	-	0.232 ± 0.117	-	-
124	2-Methyl-1-Phenylpropene	768-49-0	94	117, 132, 115	Alkenes	-	-	-	-	0.134 ± 0.120	-	-	-	-	-	-	-
125	2-Octanol	123-96-6	92	45, 55, 43	Alcohols	-	-	-	-	0.348 ± 0.078	-	-	-	-	-	-	-
126	2-Methoxy-3-Methylbutane	62016-49-3	91	59, 58, 43	Alkanes	-	-	-	-	1.717 ± 0.273	-	-	-	-	-	-	-
127	2-Ethylhexan-1-ol	104-76-7	95	57, 70, 83	Alcohols	0.853 ± 0.115	-	0.251 ± 0.049	-	1.143 ± 0.138	-	-	-	1.984 ± 0.435	1.532 ± 0.206	0.138 ± 0.030	0.919 ± 0.142
128	Formic acid	64-18-6	96	46, 45, 44	Acids	-	-	-	-	0.575 ± 0.359	-	-	-	-	-	-	-
129	3-Furanmethanol	4412-91-3	96	98, 77, 97	Alcohols	-	-	0.081 ± 0.023	-	0.375 ± 0.003	-	-	-	-	-	-	-
130	3,4-Dimethylbenzaldehyde	5973-71-7	96	133, 134, 105	Aldehydes	-	-	-	-	0.342 ± 0.007	-	-	-	-	-	-	-
131	Bis(2-ethylhexyl) adipate	103-23-1	94	129, 70, 147	Esters	-	-	-	-	0.642 ± 0.147	-	-	-	-	-	-	-
132	(+)-alpha-Pinene	7785-70-8	100	93, 91, 92	Alkenes	-	-	0.402 ± 0.044	-	-	-	-	-	-	-	0.763 ± 0.320	-
133	Sabinene	3387-41-5	97	93, 91, 69	Alkenes	-	-	-	-	-	-	-	-	-	-	0.141 ± 0.048	-
134	(+)-3-Carene	498-15-7	94	93, 91, 77	Alkenes	-	-	-	-	-	-	-	-	-	-	0.054 ± 0.016	-
135	Beta-pinene	18172-67-3	100	93, 69, 41	Alkenes	0.380 ± 0.014	-	0.549 ± 0.077	-	-	-	-	-	-	-	1.428 ± 0.256	-
136	3-Carene	13466-78-9	99	93, 91, 79	Alkenes	0.290 ± 0.122	-	0.234 ± 0.023	-	-	-	-	-	-	-	0.800 ± 0.142	-
137	2-Octen-4-one	4643-27-0	98	69, 41, 84	Ketones	-	-	-	-	-	-	-	-	-	-	0.317 ± 0.103	-
138	1-Pentanol	71-41-0	95	42, 55, 70	Alcohols	-	-	-	-	-	-	-	-	-	-	0.098 ± 0.012	0.274 ± 0.275
139	2,6-Dimethyl-4-heptanone	108-83-8	93	85, 57, 41	Ketones	-	-	-	-	-	-	-	-	-	-	0.124 ± 0.005	-
140	3,4-Dimethyl-2,4,6-octatriene	57396-75-5	94	121, 105, 136	Alkenes	-	-	-	-	-	-	-	-	-	-	0.058 ± 0.011	-
141	(4E,6Z)-2,6-Dimethylocta-2,4,6-triene	7216-56-0	94	121, 105, 136	Alkenes	-	-	-	-	-	-	-	-	-	-	0.039 ± 0.006	-
142	Formic acid, heptyl ester	112-23-2	95	70, 55, 56	Esters	-	-	-	-	-	-	-	-	-	-	0.054 ± 0.010	-
143	2-Methyl-2-dodecanol	1653-37-8	95	59, 43, 41	Alcohols	-	-	-	-	-	-	-	-	-	-	0.229 ± 0.044	-
144	4-Ethylcyclohexanol	4534-74-1	97	81, 84, 58	Alcohols	-	-	-	-	-	-	-	-	-	-	0.053 ± 0.012	0.582 ± 0.019
145	1-Octanol	111-87-5	98	56, 55, 69	Alcohols	-	-	-	-	-	-	-	-	-	0.169 ± 0.009	0.075 ± 0.018	0.403 ± 0.012
146	2-Butylpyridine	5058-19-5	97	93, 106, 120	Others	-	-	-	-	-	-	-	-	-	-	0.135 ± 0.032	0.765 ± 0.018
147	(E)-para-2-menthen-1-ol	29803-81-4	95	43, 93, 139	Alcohols	-	-	-	-	-	-	-	-	-	-	0.033 ± 0.008	-
148	2-(4-Methylenecyclohexyl)propan-2-yl acetate	93836-50-1	96	93, 43, 136	Esters	-	-	-	-	-	-	-	-	-	-	0.085 ± 0.020	-
149	(E)-citral	141-27-5	98	69, 41, 84	Aldehydes	-	-	-	-	-	-	-	-	-	-	0.135 ± 0.034	-
150	alpha-Curcumene	644-30-4	99	119, 132, 105	Alkenes	-	-	-	-	-	-	-	-	-	0.254 ± 0.005	0.206 ± 0.043	-
151	Ethyl cinnamate	103-36-6	100	131, 103, 176	Esters	-	-	3.933 ± 1.251	-	-	-	-	-	-	-	0.978 ± 0.175	-
152	m-Xylene	108-38-3	91	91, 106, 105	Alcohols	-	-	-	-	-	-	-	-	0.257 ± 0.011	0.221 ± 0.015	-	-
153	m-cymene	535-77-3	95	119, 134, 91	Aromatics	0.448 ± 0.324	-	-	-	-	-	-	-	0.294 ± 0.279	-	-	-
154	(+)-4-Carene	29050-33-7	97	93, 121, 136	Alkenes	-	-	-	-	-	-	-	-	0.120 ± 0.054	-	-	-
155	Acetic acid, 2-ethylhexyl ester	103-09-3	95	43, 70, 57	Esters	-	-	-	-	-	-	-	-	0.116 ± 0.080	-	-	-
156	Phytane	638-36-8	97	71, 85, 57	Alkanes	-	-	-	-	-	-	-	-	0.096 ± 0.040	-	-	-
157	Naphthalene	91-20-3	95	128, 127, 129	Others	-	-	-	-	-	-	-	-	0.451 ± 0.044	-	-	-
158	Tetracosane	646-31-1	98	57, 71, 85	Alkanes	0.629 ± 0.134	-	-	-	-	-	-	-	0.321 ± 0.101	-	-	0.798 ± 0.133
159	Benzothiazole	95-16-9	93	135, 108, 69	Others	-	-	-	-	-	-	-	-	0.304 ± 0.055	0.274 ± 0.020	-	-
160	3,5-Dimethyloctane	15869-93-9	96	57, 43, 71	Alkanes	-	-	-	-	-	-	-	-	-	-	-	0.283 ± 0.050
161	5-Methyl-5-propylnonane	17312-75-3	97	71, 57, 85	Alkanes	-	-	-	-	-	-	-	-	-	-	-	0.499 ± 0.068
162	2-Pentylthiophene	4861-58-9	90	97, 154, 98	Others	-	-	-	-	-	-	-	-	-	-	-	0.325 ± 0.226
163	Didecyl ether	2456-28-2	93	57, 71, 70	Ethers	-	-	-	-	-	-	-	-	-	-	-	0.140 ± 0.090
164	1-Heptanol	111-70-6	95	70, 56, 55	Alcohols	-	-	-	-	-	-	-	-	-	-	-	0.421 ± 0.006
165	Octacosane	630-02-4	96	57, 71, 85	Alkanes	0.171 ± 0.041	-	-	-	-	-	-	-	-	-	-	0.180 ± 0.057
166	Bornyl acetate	5655-61-8	95	95, 93, 121	Esters	-	-	0.098 ± 0.013	-	-	-	-	-	-	0.141 ± 0.096	-	-
167	Dimethyltetrasulfane	5756-24-1	93	158, 79, 80	Sulfide	-	-	-	-	-	-	-	-	-	0.798 ± 0.341	-	-
168	Geranylacetone	3796-70-1	93	43, 69, 136	Ketones	-	-	-	-	-	-	-	-	-	0.227 ± 0.026	-	-
169	Dimethyl phthalate	131-11-3	93	163, 77, 164	Esters	-	-	-	-	-	-	-	-	-	0.258 ± 0.011	-	-
170	(E)-2-methoxy-4-(prop-1-enyl)phenol	5932-68-3	95	164, 149, 131	Phenols	-	-	-	-	-	-	-	-	-	0.235 ± 0.025	-	-
171	Acetone	67-64-1	98	43, 58, 44	Ketones	-	-	0.263 ± 0.057	-	-	-	-	-	-	-	-	-
172	1,3,3-trimethyltricyclo[2.2.1.02,6]heptane	488-97-1	99	93, 91, 92	Alkanes	-	-	0.205 ± 0.051	-	-	-	-	-	-	-	-	-
173	2,6-Dimethylpyrazine	108-50-9	97	108, 42, 40	Pyrazine	-	-	0.165 ± 0.061	-	-	-	-	-	-	-	-	-
174	3-Ethyl-2,5-diMethylpyrazine	13360-65-1	93	135, 136, 108	Pyrazine	-	-	0.125 ± 0.019	-	-	-	-	-	-	-	-	-
175	2,6,6-Trimethyl-2,4-cycloheptadien-1-one	503-93-5	93	107, 91, 108	Ketones	-	-	0.050 ± 0.011	-	-	-	-	-	-	-	-	-
176	(1R,2R,5S)-5-Methyl-2-(prop-1-en-2-yl)cyclohexanol	29141-10-4	94	67, 81, 71	Alcohols	-	-	0.017 ± 0.003	-	-	-	-	-	-	-	-	-
177	4-n-propylanisole	104-45-0	97	121, 150, 122	Ethers	-	-	0.236 ± 0.043	-	-	-	-	-	-	-	-	-
178	2,4-Cyclohexadiene-1-methanol	1686-20-0	94	94, 59, 79	Alcohols	-	-	0.059 ± 0.016	-	-	-	-	-	-	-	-	-
179	Citral	5392-40-5	94	69, 41, 84	Aldehydes	1.234 ± 0.187	-	0.053 ± 0.013	-	-	-	-	-	-	-	-	-
180	trans-Calamenene	73209-42-4	94	159, 160, 202	Alkenes	-	-	0.122 ± 0.020	-	-	-	-	-	-	-	-	-
181	3-Phenylpropanol	122-97-4	91	117, 91, 118	Alcohols	-	-	0.108 ± 0.042	-	-	-	-	-	-	-	-	-
182	Cinnamyl acetat	103-54-8	91	115, 43, 133	Esters	-	-	0.032 ± 0.017	-	-	-	-	-	-	-	-	-
183	2,6,10-Trimethyldodecane	3891-98-3	98	71, 57, 85	Alkanes	0.207 ± 0.145	-	-	-	-	-	-	-	-	-	-	-
184	Acetic acid, 1,7,7-trimethyl-bicyclo [2.2.1]hept-2-yl ester	92618-89-8	93	95, 93, 43	Acids	0.052 ± 0.056	-	-	-	-	-	-	-	-	-	-	-
185	Tetratetracontane	7098-22-8	91	57, 43, 85	Alkanes	0.190 ± 0.029	-	-	-	-	-	-	-	-	-	-	-
186	Neral	106-26-3	96	69, 41, 109	Aldehydes	0.559 ± 0.077	-	-	-	-	-	-	-	-	-	-	-

“-” Not detected.

## Data Availability

The original contributions presented in this study are included in the article. Further inquiries can be directed to the corresponding authors.
